# Tomonaga–Luttinger liquid and quantum criticality in spin-$\frac{1}{2}$ antiferromagnetic Heisenberg chain *C*_14_*H*_18_*CuN*_4_*O*_10_ via Wilson ratio

**DOI:** 10.1093/pnasnexus/pgae363

**Published:** 2024-08-23

**Authors:** Sharath Kumar Channarayappa, Sankalp Kumar, N S Vidhyadhiraja, Sumiran Pujari, M P Saravanan, Amal Sebastian, Eun Sang Choi, Shalinee Chikara, Dolly Nambi, Athira Suresh, Siddhartha Lal, D Jaiswal-Nagar

**Affiliations:** School of Physics, IISER Thiruvananthapuram, Thiruvananthapuram, Kerala 695551, India; Department of Physics, Indian Institute of Technology Bombay, Mumbai, Maharashtra 400076, India; Theoretical Sciences Unit, Jawaharlal Nehru Center for Advanced Scientific Research, Bengaluru, Karnataka 560064, India; Department of Physics, Indian Institute of Technology Bombay, Mumbai, Maharashtra 400076, India; UGC-DAE Consortium for Scientific Research, Indor, Madhya Pradesh 452001, India; School of Physics, IISER Thiruvananthapuram, Thiruvananthapuram, Kerala 695551, India; National High Magnetic Field Lab (NHMFL), Tallahassee, FL 32310, USA; National High Magnetic Field Lab (NHMFL), Tallahassee, FL 32310, USA; School of Physics, IISER Thiruvananthapuram, Thiruvananthapuram, Kerala 695551, India; School of Physics, IISER Thiruvananthapuram, Thiruvananthapuram, Kerala 695551, India; Department of Physical Sciences, IISER Kolkata, Mohanpur Campus, West Bengal 741246, India; School of Physics, IISER Thiruvananthapuram, Thiruvananthapuram, Kerala 695551, India

**Keywords:** quantum criticality, Tomonaga–Luttinger liquid, Wilson ratio, one-dimensional antiferromagnetic Heisenberg chain, quantum phase transition

## Abstract

The ground state of a one-dimensional spin-$\frac{1}{2}$ uniform antiferromagnetic Heisenberg chain (AfHc) is a Tomonaga–Luttinger liquid which is quantum-critical with respect to applied magnetic fields up to a saturation field $\mu_{0}H_{s}$ beyond which it transforms to a fully polarized state. Wilson ratio has been predicted to be a good indicator for demarcating these phases [Phys. Rev. B 96, 220401 (2017)]. From detailed temperature and magnetic field-dependent magnetization, magnetic susceptibility and specific heat measurements in a metalorganic complex and comparisons with field theory and quantum transfer matrix method calculations, the complex was found to be a very good realization of a spin-$\frac{1}{2}$ AfHc. Wilson ratio obtained from experimentally obtained magnetic susceptibility and magnetic contribution of specific heat values was used to map the magnetic phase diagram of the uniform spin-$\frac{1}{2}$ AfHc over large regions of phase space demarcating Tomonaga–Luttinger liquid, saturation field quantum critical, and fully polarized states. Luttinger parameter and spinon velocity were found to match very well with the values predicted from conformal field theory.

**Significance Statement**

This work presents an experimental phase diagram of a spin-$\frac{1}{2}$ antiferromagnetic Heisenberg chain, utilizing Wilson ratio as a robust indicator of quantum criticality in Tomonaga–Luttinger liquids. Through comprehensive measurements in single crystals of novel material $C_{14}H_{18}CuN_{4}O_{10}$, distinct phases of Tomonaga–Luttinger liquid, quantum critical and fully polarized phase are elucidated across a wide parameter space. Very good agreement between theory and experiment validates $C_{14}H_{18}CuN_{4}O_{10}$ as an exceptional realization of spin-$\frac{1}{2}$ antiferromagnetic Heisenberg chain system. Our findings highlight the broader impact of systems with low saturation fields in studying quantum critical phenomena and underscore the significance of Wilson ratio in characterizing such systems. This work advances understanding in one-dimensional quantum systems and opens avenues for exploring emergent quantum phenomena across diverse materials.

## Introduction

Phase diagrams provide a comprehensive means to study the complex behavior of systems near phase transitions, bridging theoretical predictions with experimental observations and enhancing our understanding of emergent phenomena. A text book example is the pressure–temperature phase diagram of water that has a line of first-order phase transitions that terminate into a critical point of $P_{c}$ = 221 bar and $T_{c}$ = 374°C (1, 2). An analogous phase diagram was recently observed in a frustrated two-dimensional spin-$\frac{1}{2}$ magnet, $SrCu_{2}(BO_{3}{)}_{2}$ (3). When the finite temperature critical point is suppressed to T=0K, a quantum critical point (QCP) associated with a quantum phase transition (QPT) emerges (4, 5). A QCP is expected to affect a finite portion of the phase diagram in a cone-like region bounded by the entanglement temperature (6). However, very few examples of phase diagrams exhibiting the quantum critical cone along-with the phases associated with the QCP, exist in the literature, the prominent being the phase diagram of the heavy fermion $YbRh_{2}Si_{2}$ (7). The cone-like quantum critical (QC) region is demarcated by boundaries that are determined by the condition $k_{B}T\propto |r-r_{c}{|}^{\nu z}$, where *ν* and *z* denote the correlation length critical exponent and dynamic critical exponent, respectively that are usually universal (4, 5). The nonthermal control parameter *r* that is used to tune the QCP are pressure, doping, magnetic field, etc. In this regard, magnetic field turns out to be a very useful handle to probe quantum criticality in diverse systems due to the ease of application of a magnetic field to reversibly and continuously tune a system towards a QCP (7–11). However, large values of magnetic fields needed to tune a QCP in systems having large exchange coupling constants make experimental investigations of phase diagrams difficult in quantum critical systems (12–15). Therefore, systems having low values of exchange coupling constants in which the QCP could be tuned by low values of applied magnetic fields easily accessible by laboratory magnets provide excellent platforms using which the complexities associated with QPT’s could be studied and phase diagrams made. Our work describes a detailed and extended phase diagram of a quantum critical system with quite favorable coupling constant.

QPT’s have been observed in diverse and complex systems ranging from heavy fermions to high temperature superconductors (7, 16, 17) but have not yet been understood completely. A study of QPT’s in systems that are realizations of exactly solvable models can offer deeper insights into this phenomenon. One such exactly solvable model is the spin-$\frac{1}{2}$ one-dimension (1D) antiferromagnetic Heisenberg chain (AfHc) that can be described by a relativistic field theory in the low energy limit. Specifically, the Tomonaga–Luttinger liquid (TLL) theory, a relativistic free boson field theory, is known to describe the ground state of a spin-$\frac{1}{2}$ AfHc (18). In this theory, the velocity of the spin-waves *u* is a variable unlike the speed of light in a true relativistic theory which is a constant (19). This and another parameter of the TLL theory, namely, the Luttinger parameter *K*, can be determined in an integrable model (20) and can fully describe the low-energy features within the TLL framework. For free fermions K=1, while K<1 and K>1 represent repulsive and attractive interactions, respectively. It is anticipated that *K* would change continuously for the simple spin-$\frac{1}{2}$ AfHc, from K=0.5 in the zero field to K=1 at saturation magnetic field $\mu_{0}H_{s}$ (20).

**Fig. 1.**
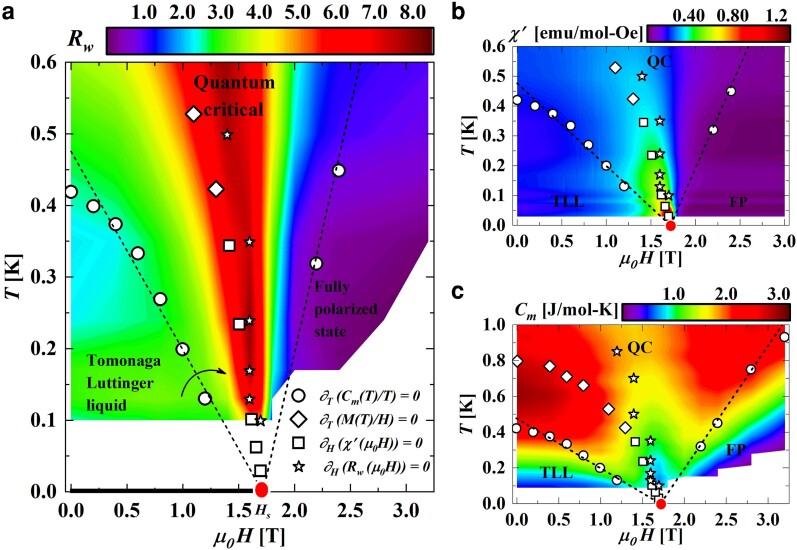
a) Temperature-magnetic field phase diagram of CuD displaying Tomonaga–Luttinger liquid, quantum critical cone and Fully polarized phases. The T=0 K line of critical points has been shown by a thick black line that terminates in a quantum critical saturation field end point, $\mu_{0}H_{s}$, shown by a red filled dot. Wilson ratio, $R_{w}(\mu_{0}H,T)$, is shown on the contour plot with the color scale shown at the top where dark red denotes the highest value and dark blue denotes the least value. Open circles represent the maxima of $C_{m}(T)/T$ while the maxima of the M(T)/H and $\chi^{\prime }(\mu_{0}H)$ are shown by open diamonds and open squares, respectively. The peak in $R_{w}(\mu_{0}H)$ (see Fig. S5) is shown as open stars. The black dashed lines represent fits to Eq. 4 with $\alpha_{1}$ and $\alpha_{2}$ as fit parameters which were obtained as 3.61 and 1.45 respectively. Curved arrow represents the cross-over from z=1 to z=2 state. (b) and (c) represent the phase diagram made using $\chi^{\prime }(\mu_{0}H,T)$ and $C_{m}(\mu_{0}H,T)$ respectively. In (b), 15 scans of temperature with field-dependent $\chi^{\prime }(\mu_{0}H)$ ($\mu_{0}H⊥$  *a*-axis) were used to create the contour plot. In (c), 16 scans of magnetic fields with temperature-dependent $C_{m}(T)$ ($\mu_{0}H⊥$  *a*-axis) were used to create the contour plot.

The Hamiltonian of the spin-$\frac{1}{2}$ AfHc in a magnetic field *H* is given by:


(1) $$H=J\sum_{i}{\vec{S}}_{i}\cdot {\vec{S}}_{i+1}-g\mu_{B}H\sum_{i}{\vec{S}}_{i}^{z}$$

where *J* is the exchange coupling constant in the chain. At T=0 K and $\mu_{0}H=0$ T, the spin-$\frac{1}{2}$ AfHc fails to develop any long range order, however, the correlation functions exhibit an algebraic decay making the AfHc a quantum critical system. The spin-$\frac{1}{2}$ AfHc is also critical with respect to an applied field up to the saturation field $H_{s}$, given by $g\mu_{B}m_{s}H_{s}=J$ (21) above which it transforms to a fully polarized (FP) state as shown in Fig. 1 which is an exact eigenstate of the Hamiltonian Eq. 1 different from TLL. So, $H_{s}$ marks the end-point of a line of QCP’s separating the FP state from the partially magnetized TLL state at lower fields (22). The excitation’s of the TLL are spinons that are topological excitation’s in the spin order and form a continuum excitation spectrum over an extended range of energy and momentum (14). In contrast, the excitation spectrum of the FP state comprises gapped excitation’s (23).

Wilson ratio, $R_{w}$, defined as the ratio of magnetic susceptibility $\chi^{\prime }$ to specific heat *C* divided by temperature *T* (24, 25):


(2) $$R_{w}=\frac{4}{3}{\left(\frac{\pi k_{B}}{g\mu_{B}}\right)}^{2}\frac{\chi^{\prime }}{C/T}$$

is proposed to be an important parameter for characterizing the TLL phase boundary as well as the QC region associated with the saturation field critical point (23). In Eq. 2, $k_{B}$, $\mu_{B}$, and *g* denote Boltzmann constant, Bohr magneton, and Landé factor, respectively. $R_{w}=1$ for a system of noninteracting fermions (26) and equals 2 in the Kondo regime of the impurity problem (25). Wilson ratio quantifies interaction effects and spin fluctuations in a strongly correlated system (27, 28). For instance, $R_{w}$ was found to have a value close to 8 in the vicinity of the QCP in a strongly correlated layered cobalt oxide $BiBa_{0.66}K_{0.36}O_{2}CoO_{2}$ (29). Even though the magnetic field variation of $R_{w}$ was studied in this work, an experimental phase diagram using $R_{w}$ as contours was not made. Wilson ratio is expected to be in the range $0<R_{w}<10$ across the phase diagram associated with a TLL (23).

There are several materials, for instance $KCuF_{3}$ and $Sr_{2}CuO_{3}$, that are good realizations of a spin-$\frac{1}{2}$ AfHc. However, their large values of exchange coupling constants, $J/k_{B}∼$380 K in $KCuF_{3}$ (14, 30–33) and 2,200 K in $Sr_{2}CuO_{3}$ (15), make the corresponding saturation field $\mu_{0}H_{s}$ extremely high at hundreds and thousands of Teslas. The most well-studied spin-$\frac{1}{2}$ AfHc is $Cu.(C_{4}H_{4}N_{2}).(NO_{3}{)}_{2}$ (CuPzN) that has a moderate $J/k_{B}$ of ∼10.3 K (12, 13, 34) making $\mu_{0}H_{s}∼$14 T. This makes the investigation of the FP state in CuPzN difficult using lab magnets. Additionally, CuPzN undergoes a 3D ordering at 0.107 K (35) complicating the investigation of the thermodynamics of the spin-$\frac{1}{2}$ AfHc.

In this work, we have investigated the thermodynamics of the spin-$\frac{1}{2}$ AfHc over a large range of parameters from $\mu_{0}H=0$ T till saturation field $\mu_{0}H_{s}$ and far above $\mu_{0}H_{s}$ (FP state) in a new spin-$\frac{1}{2}$ AfHc compound copper bisoxalate amminopyridate $\left(C_{5}H_{7}N_{2}{)}_{2}[Cu(C_{2}O_{4}{)}_{2}].2(H_{2}O\right)$ having the formula $C_{14}H_{18}CuN_{4}O_{10}$ and abbreviated as CuD, that has a $J/k_{B}$ value of ∼1.23 K and a corresponding saturation field $\mu_{0}H_{s}$ of ∼1.7 T (36). Magnetization normalized by field M(T)/H, magnetic susceptibility $\chi^{\prime }(\mu_{0}H)$ and magnetic contribution of specific heat $C_{m}(T)$ curves generated using quantum transfer matrix calculations are found to fit the experimentally produced data excellently over a large range of temperatures and field values. Magnetic susceptibility and magnetic contribution of specific heat were used to calculate the Wilson ratio using which a complete phase diagram of a spin-$\frac{1}{2}$ Heisenberg antiferromagnetic chain has been drawn experimentally for the first time marking the Tomonaga–Luttinger liquid, quantum critical and FP phases. The quantum critical phase boundaries corresponding to the saturation field quantum critical point were found to extend to a large portion of the phase diagram unlike CuPzN where deviations were observed due to a 3D ordering. We observed excellent data collapse in thermodynamic properties arising due to the quantum critical scaling. Finally, we obtained spinon velocity *u* and the Luttinger parameter *K* experimentally whose values were found to match perfectly with theoretical predictions (20). The sign of the parameter *K* revealed that the strength and nature of spinon interactions are repulsive, whereas the spinon velocity *u* revealed that the dynamics are low-energy. With a $J/k_{B}$ of 0.106 meV, the spinon bandwidth is expected to be ∼0.16 meV in CuD. This small value of the bandwidth and observations from the transport measurements which indicate that CuD is a robust insulator, the charge channel with holon excitation is expected to be negligible in CuD.

CuD crystallizes in the monoclinic crystal structure (P21/c) with lattice parameters a=3.7064  Å, b=20.2976  Å and c=11.9059  Å. The structure comprises $Cu^{2+}$ ions that coordinate with four O atoms in the basal plane to form a square planar geometry. The $[Cu(C_{2}O_{4}){]}^{-2}$ ions link together, forming a straight $Cu^{2+}$ chain along the crystallographic *a*-axis as shown in Fig. S1. This chain has a regular Cu⋯Cu spacing of 3.706 Å, formed by corner-sharing oxygen atoms of the $CuO_{6}$ octahedra (36). To investigate the thermodynamics of CuD, external magnetic field was applied perpendicular to the chain direction. As is well known, the excitation spectrum of a TLL comprises multispinons continuum that is isotropic in the absence of any field (18, 20, 37, 38). However, on field application, the spin correlations become anisotropic, such that $S_{xx}(q,w)=S_{yy}(q,w)\neq S_{zz}(q,w)$. Therefore, the maximum effect to the total spectrum arises from the tranverse contributions where a significant loss of spectral weight arises at the center of the Brillouin zone when compared to the longitudinal spectrum (39). Accordingly, the field was applied perpendicular to the chain direction ($\mu_{0}H⊥$ crystallographic *a*-axis) since the thermodynamics of a spin-$\frac{1}{2}$ AfHc in a field is determined mainly by the transverse excitation modes (39–41).

The thermodynamics of the integrable spin-$\frac{1}{2}$ AfHc was calculated by considering a quasi-linear energy-momentum dispersion of the spinons having a velocity *u* using which the free energy equations are solved analytically and implemented numerically using the quantum transfer matrix (QTM) method (see Supplementary Material, section 6 for details). The field-theoretic spinless fermionic description of the TLL gives the following scaling form of the free energy


(3) $$F(T,\mu )=\frac{J-2g\mu_{B}H}{4}+\frac{(k_{B}T{)}^{3/2}}{\sqrt{J}}\Phi \left(\frac{g\mu_{B}(H_{s}-H)}{k_{B}T}\right)$$

at leading order. The first term is the ground state energy denoting the FP state, while the second term denotes the asymptotic scaling behavior close to the QCP arising from the thermal population of spinons. Higher order corrections come from spinon interactions that are irrelevant in the renormalization group sense and will be sub-dominant.

## Results and Discussion

The magnetic phase diagrams of CuD produced using Wilson ratio ($R_{w}$), magnetic susceptibility ($\chi^{\prime }$), and magnetic contribution of the specific heat ($C_{m}$) are shown in Fig. 1a, b, and c, respectively. The contour plot made using $R_{w}$, distinguishes three distinct regions: a gapless Tomonaga–Luttinger liquid region, a quantum critical cone and the gapped FP region. The ground state wave-function of the TLL containing entangled spins evolves continuously with the application of field such that at the saturation field $\mu_{0}H_{s}$, the TLL breaks down and an unentangled FP state appears on the right side of $\mu_{0}H_{s}$ (6). It has been reported that Wilson ratio and specific heat is a better marker of the TLL phase boundary (23) compared to the magnetization/magnetic susceptibility measurements. Accordingly, in Fig. 1a the region below the open circles between $0\leq \mu_{0}H\leq \mu_{0}H_{s}$ marked by maxima in $C_{m}(T)/T$ (see Fig. S4a) has been marked as the TLL phase. The contour plots of the Wilson ratio suggest that the TLL phase is subdivided into two regions (see Fig. 1a), which is not evident from the susceptibility contours of the same color, Fig. 1b, but can be inferred from the specific heat contours that also exhibit gradients in values within the TLL phase (Fig. 1c).

The FP state is also marked by specific heat measurements as temperatures in $C_{m}(T)/T$ plots (see Fig. S4b) where an exponential drop occurs in the specific heat. From Fig. 1a, it can be seen that the Wilson ratio $R_{w}$ falls to values less than 0.5 in this region. Magnetization also falls to a low value in this region (see Fig. 1b), while specific heat remains finite and with variable magnitude (see Fig. 1c). However, the Wilson ratio has a small and uniform contour in the FP state, demonstrating the usefulness of the Wilson ratio as an indicator for mapping the phase diagram of a quasi-one dimensional spin-$\frac{1}{2}$ Heisenberg antiferromagnet.

The universal scaling behavior associated with the quantum critical endpoint $\mu_{0}H_{s}$ and signaling a breakdown of the TLL phase (42) is captured by the quantum critical phase boundaries. These phase boundaries are given by the expression (23):


(4) $$T_{\mathrm{spinon}}=\frac{g\mu_{B}\mu_{0}}{\alpha_{1}k_{B}}(H_{s}-H);\;T_{\mathrm{magnon}}=-\frac{g\mu_{B}\mu_{0}}{\alpha_{2}k_{B}}(H_{s}-H)$$

where $T_{\mathrm{spinon}}$ and $T_{\mathrm{magnon}}$ represent the left and right lines of the quantum critical cone governed by the spinon excitations of the TLL phase and magnon excitations of the FP phase, respectively. $\alpha_{1}$ and $\alpha_{2}$ are constants. A comparison of the Eq. 4 with the quantum critical phase boundaries $k_{B}T\propto |r-r_{c}{|}^{\nu z}$ yields νz=1. It is known that correlation-length exponent $\nu =\frac{1}{2}$ and dynamical exponent z=2 at the saturation field $\mu_{0}H_{s}$ of a spin-$\frac{1}{2}$ AfHc (18, 23, 38). So, the phase boundaries associated with the saturation field critical point of a spin-$\frac{1}{2}$ AfHc is governed by the dynamic critical exponent z=2. The theoretical phase boundaries (black dashed lines of Fig. 1a) are found to follow the experimental data points in CuD to a very large portion of the phase diagram and not limited to areas close to $\mu_{0}H_{s}$, in contrast to CuPzN (23, 43) where deviations are observed due to 3D ordering in CuPzN. Additionally, since in the TLL phase z=1 (4), the left QC line governs the transformation of z=1 to z=2 as shown by thick curved arrow in Fig. 1a. On the other hand, the right QC line demonstrates the transformation of the field induced gap, *Δ*, that goes linearly with field, $\Delta \propto \mu_{0}(H-H_{s})$ (21), to the quantum critical region.

**Fig. 2.**
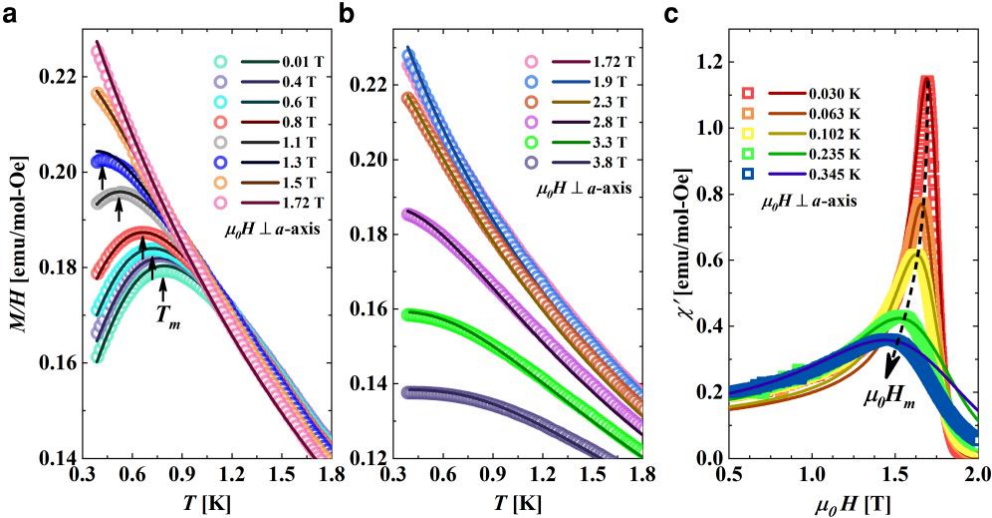
a) At various fields up to $\mu_{0}H_{s}$, M(T)/H, is shown by open colored circles. The corresponding colored solid lines show the quantum transfer matrix theoretical calculations. The peak temperature denoted by $T_{m}$ is represented by black arrows. b) Colored open circles and corresponding colored solid lines represent M(T)/H and quantum transfer matrix calculations respectively at saturation field $\mu_{0}H_{s}$ and above. c) Open triangles represent the field variation of $\chi^{\prime }(\mu_{0}H)$, at different values of temperatures while the solid lines of the same color denote the result of QTM calculations. Black dashed line is a guide to the eye and indicates the shift of the maxima.

**Fig. 3.**
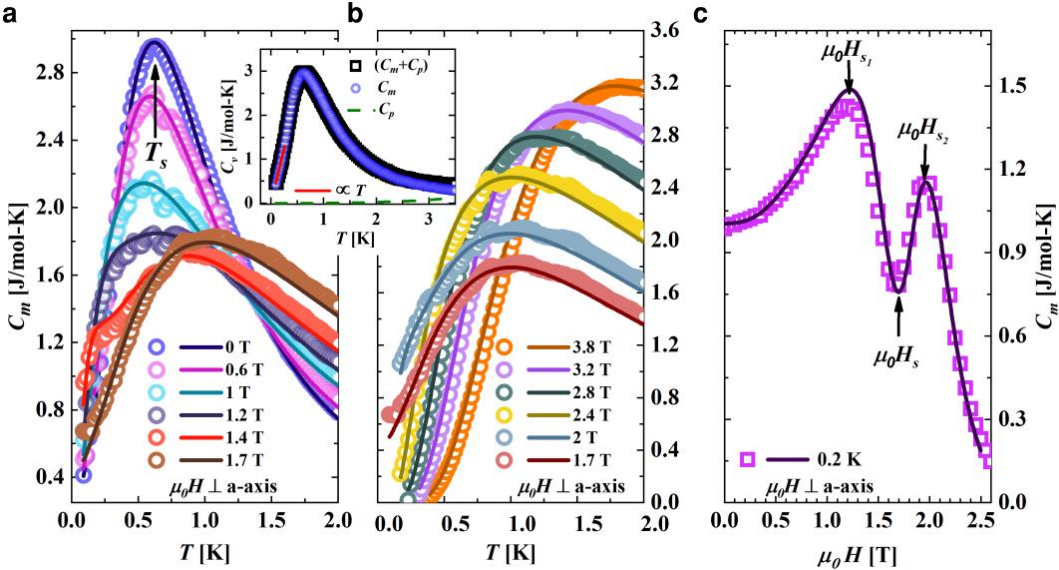
Black open squares in inset of (a and b) represent the actual measured $C_{v}(T)$ while the green dashed line represents the estimated $C_{p}(T)$ to low temperatures from the high temperature Debye-Einstein model fit. $C_{m}(T)$, illustrated as blue open circles, is obtained by subtracting $C_{p}(T)$ from $C_{v}(T)$. At low temperatures, red solid line is a linear fit to $C_{m}(T)$ obtained for $\mu_{0}H=0$ T. a) Open colored circles represent the temperature variation of $C_{m}(T)$ at various applied magnetic fields up to $\mu_{0}H_{s}$, and b) at $\mu_{0}H_{s}$ and above. The corresponding QTM calculations are represented by solid curves of the same color. c) Purple open circles represent the magnetic field variation of $C_{m}$ at T=0.2 K while the solid line is the result of QTM calculations. Arrows indicate the position of the two maxima and a QCP.

Magnetization, magnetic susceptibility and specific heat measurements used to make the phase diagram of Fig. 1 are shown in Fig. 2 and Fig. 3. Colored open circles in Fig. 2a and b depicts the magnetic field evolution of temperature-dependent magnetization normalized to field, M(T)/H at fields below (Fig. 2a) and above (Fig. 2b) the saturation field $\mu_{0}H_{s}$. The corresponding solid lines in Fig. 2a and b denote the result of QTM calculations done with only one free parameter, *J*. The calculated M(T)/H curves were obtained as a function of $T/J/k_{B}$. It can be seen that the calculated curves fall exactly on top of the experimentally obtained ones once the temperature axis is scaled by the experimentally obtained value of $J/k_{B}=1.23$ K indicating that CuD is an excellent realization of a spin-$\frac{1}{2}$ AfHc.

The low field curves are characterized by a peak demarcated as $T_{m}$ (open diamonds of Fig. 1) indicating the cross-over from a TLL to QC. $T_{m}$ is predicted to occur at $T_{m}∼0.641J$ with the maximum value $(M(T)/H{)}_{\max}∼0.146Ng^{2}\mu_{B}^{2}/J$ in the limit of $\mu_{0}H\to 0$ (28, 44). From the experimentally obtained value of $T_{m}=0.79$ K and $(M(T)/H{)}_{\max}=0.18$ emu/mol*Oe at the lowest applied field of 0.01 T, $J/k_{B}$ is obtained as 1.23 K while the Landé g-factor, *g*, is obtained as 2.03. These numbers exactly match the values obtained from fitting the uniform spin-$\frac{1}{2}$ AfHc model (high temperature series expansion) to the 0.01 T data (see Supplementary Material, section 2 and Fig. S2). Using $J/k_{B}=1.23$ K and g=2.03, $\mu_{0}H_{s}$ is obtained as 1.8 T. From Fig. 2a, it is to be noted that as the applied field increases, $T_{m}$ steadily decreases such that for a field of 1.5 T, it falls below the lowest measurable temperature of 0.38 K. At fields near $\mu_{0}H_{s}$, M(T)/H exhibits diverges as T→0 K, indicating quantum criticality (18, 41). For fields above $\mu_{0}H_{s}$, the magnetization plateaus at low temperatures where the ground state is a gapped, field-induced polarized state (18, 41).

For fields at which $T_{m}$ fell below the measurable temperatures of 0.38 K (lowest attainable temperature of SQUID magnetometer), it was difficult to ascertain the TLL phase boundary, especially for fields closer to $\mu_{0}H_{s}$. To overcome this problem, field-dependent magnetic susceptibility $\chi^{\prime }(\mu_{0}H)$ measurements were performed at different temperatures up to 30 mK using an ac susceptometer and shown as colored open squares in Fig. 2c. The curves present a peak at magnetic field $\mu_{0}H_{m}$ (open squares in Fig. 1) while the corresponding solid curves are the result of QTM calculations obtained in the way described above. As can be observed, the match between the calculated theory curves and the experimentally obtained curves is very good. The peak magnetic field $\mu_{0}H_{m}$ is found to shift to lower magnetic fields with an increase in temperature due to increased thermal fluctuations.

The contour plot of Fig. 1c was made using specific heat measurements shown in Fig. 3a and b. Temperature dependence of magnetic specific heat, $C_{m}(T)$, shown in Fig. 3a and b was obtained by subtracting the phonon contribution, $C_{p}(T)$, shown as green dashed line in the inset of Fig. 3a and b from the total specific heat $C_{v}(T)$ (black open squares in inset of Fig. 3a and b). $C_{p}(T)$ was calculated using the Debye-Einstein model having one and two terms, respectively (see Supplementary Material, Section 3 and Fig. S3 for details). Although phonons start to contribute at 1.2 K and above (green dashed line), the nuclear contribution to $C_{m}(T)$ is relevant only at ultra-low temperatures of 100 mK and below as well as high magnetic fields of the order of 10 T, and hence, can be neglected in our measured range of temperatures and magnetic fields. The blue open circles in the insets of Fig. 3a and b represent the zero field $C_{m}(T)$, which indicates the absence of long-range ordering (LRO) down to 100 mK.

Temperature variation of $C_{m}(T)$ is shown in Fig. 3a–b for various values of applied magnetic field up to saturation field $\mu_{0}H_{s}$ (Fig. 3a) and $\mu_{0}H_{s}$ and beyond (Fig. 3b). Dark colored solid lines of the same color as the open circles show the result of QTM calculations obtained using a single free parameter, *J*. $C_{m}(T)$ was obtained as a function of $T/J/k_{B}$ and scaled to the temperature axis by normalizing it with the experimentally obtained $J/k_{B}$. As with the magnetization results above, the match between theoretically obtained and experimentally obtained curves is excellent reiterating that CuD is an excellent realization of a spin-$\frac{1}{2}$ AfHc. The curves reveal a maximum in $C_{m}(T)/T$ as the temperature is varied indicating a transformation from a TLL phase to a QC phase (see Fig. 1). At 0 T, the peak temperature of M(T)/H is nearly twice that of $C_{m}(T)/T$ since M(T)/H measures two-particle excitations while $C_{m}(T)/T$ measures one-particle density of states (41). In Fig. 3a maximum in $C_{m}(T)$ at $\mu_{0}H=0$ T is predicted to arise at $T_{s}=0.48J/k_{B}$ (28). Using the value of $J/k_{B}=1.23$ K obtained from magnetization data above, $T_{s}$ should be at 0.59 K. It is satisfying to note that $T_{s}=0.6$ K, in very good agreement with theoretical predictions.

Fig. 3a and b presents striking differences in the low temperature behavior of $C_{m}(T)$ when the applied external magnetic field is below or above the saturation field $\mu_{0}H_{s}$, respectively. While for $\mu_{0}H<\mu_{0}H_{s}$, $C_{m}(T)$ at low temperatures is finite indicating a finite density of low energy spinons, for $\mu_{0}H>\mu_{0}H_{s}$, the low temperature $C_{m}(T)$ falls to zero exponentially due to the opening up of a gap in the density of states after the transformation of the TLL state to that of a FP state. Furthermore, $C_{m}(T)$ increases linearly with temperature indicating the fermionic nature of the quasiparticles (which are spinons) of the TLL state. With an increase in the applied field, the maximum value of specific heat, $(C_{m}(T){)}_{\max}$ reduces in magnitude. The application of field results in the generation of spin strings of different lengths in the highly entangled ground state reducing the ground state’s number of spinons at the Fermi points (23, 45), and consequently, the specific heat. Fig. 3c depicts the field variation of $C_{m}(\mu_{0}H)$ at the lowest measured temperature of 0.2 K. From the plot, one can observe the presence of two maxima at $\mu_{0}H_{s_{1}}$ and $\mu_{0}H_{s_{2}}$ indicating the transformation from the TLL to a QC (at $\mu_{0}H_{s_{1}}$) and that to a fully FP state (at $\mu_{0}H_{s_{2}}$), respectively. So, at $\mu_{0}H_{s_{1}}$, quantum and thermal fluctuations reach an equal footing while at $\mu_{0}H_{s_{2}}$, fluctuations due to magnon dominate. The intermediate region between $\mu_{0}H_{s_{1}}$ and $\mu_{0}H_{s_{2}}$ denote saturation field quantum critical region where thermal fluctuations strongly couple with quantum fluctuations (23). The solid line in Fig. 3c depicts the results of QTM calculations which is seen to match very well with the experimental data to confirm once again that CuD is quite a good system to realize a spin-$\frac{1}{2}$ AfHc.

From the free energy functional of Eq. 3, magnetization *M* in the saturation field quantum critical region is found to be independent of $\mu_{0}(H-H_{s})$ (see Supplementary Material, section 7 for details) and varies as:


(5) $$M_{s}-M=g\mu_{B}\zeta (0.5)(1-\sqrt{2}){\left(\frac{k_{B}T}{4\pi J}\right)}^{\frac{1}{2}}$$

where $M_{s}$ is the saturation magnetization.

**Fig. 4.**
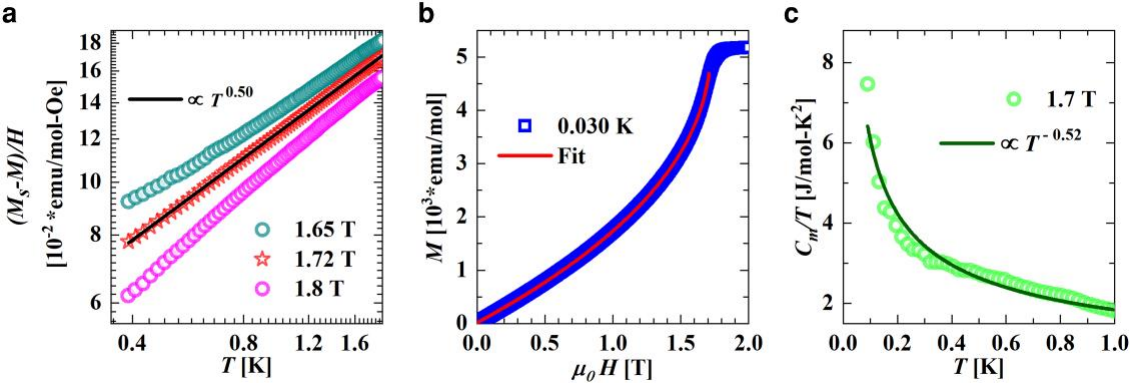
a) Log–log plot of $(M_{s}-M)/H$ as a function of temperature for fields close to the saturation field showing data for 1.65 T (olive green open circles), 1.72 T (red open stars) and 1.8 T (pink open circles). Black solid line is a straight line fit to the 1.72 T data yielding β=0.5 and $M_{s}=5,216.26$ emu/mole (see text for details). b) Blue open squares represent magnetization as a function of applied field $\mu_{0}H$ at a temperature of 0.030 K. Red solid line is a fit to the data (see text for details). c) Green open circles represent the temperature variation of $C_{m}(T)/T$ at an applied field of 1.7 T while dark green solid line is a power law fit, $T^{\alpha }$, to the data. The best fit yielded α=0.52.

The saturation field, $\mu_{0}H_{s}$, was found experimentally by fitting $(M_{s}-M)/H$ vs. *T* (obtained from Fig. 2a and b) to $T^{\beta }$ for different values of fields very close to $\mu_{0}H_{s}$ as shown in Fig. 4a. The fits were made keeping both $M_{s}$ and *β* as free parameters while having $J/k_{B}$ and *g* to have fixed values of 1.23 K and 2.03, respectively (obtained from the magnetization data analysis above). The best fit to data was obtained for an external applied field of 1.72 T that gave the value of saturation magnetization $M_{s}$ as 5,216.26 emu/mol close to the expected value of 5,212 emu/mol (see Fig. 4b), with β=0.50, identical to the expected theoretical value of $\frac{1}{2}$ from Eq. 5.

Magnetic field variation of magnetization, *M*, obtained by integrating magnetic field-dependent $\chi^{\prime }(\mu_{0}H)$ measured in CuD at T=0.030 K is shown by blue squares in Fig. 4b. It can be seen that *M* increases with field till ∼1.7 T ($\mu_{0}H_{s}$) above which it saturates due to a transformation of the TLL state to an FP state. Red solid curve in Fig. 4b represents a fit the expression $1-M/M_{s}=1.27(1-H/H_{s}{)}^{1/\delta }$ to the data between 0 T and 1.7 T considering $M_{s}=5,216.26$ emu/mol yields critical field $\mu_{0}H_{s}=1.72$ T and δ=2.2, in agreement with the predicted value of critical exponent δ=2.

From the free energy expression Eq. 3, $C_{m}(T)/T$ should diverge with temperature at the saturation field $\mu_{0}H_{s}$ since $C_{m}(T)/T$ is proportional to the density of states D(ϵ) which goes as $1/\sqrt{T}$ in one dimension. Such a divergence was indeed observed in CuD as shown in Fig. 4c where a fit of $C_{m}/T$ vs. *T* data below 1 K (green open circles) to $C_{m}/T=0.22894N_{a}k_{B}^{3/2}(JT{)}^{-\alpha }$ (dark green solid line) keeping $J/k_{B}=1.23$ K, gave a value of the exponent *α* as 0.52, very close to the expected value of 0.5 (41). The combination of power-law exponents extracted from thermodynamic measurements described above is α+β(1+δ)= 2.12, where α= 0.52, β= 0.5, and δ= 2.22, close to the theoretically expected universal scaling value of 2 (23) confirming the excellent realization of CuD as a spin-$\frac{1}{2}$ AfHc system.

**Fig. 5.**
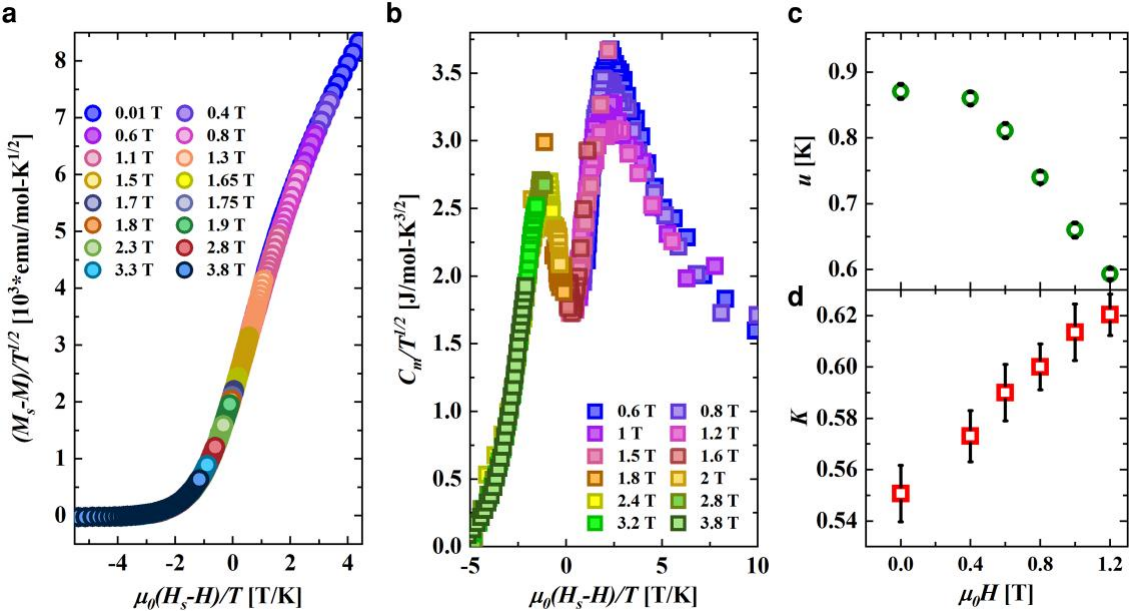
a) Filled circles represent the scaling collapses of magnetization data as described in the text with $\mu_{0}H_{s}=$ 1.72 T. b) Filled squares represent the scaling collapses of specific heat data as described in the text where $\mu_{0}H_{s}$ = 1.7 T. c) Spinon velocity *u* as a function of field calculated from $C_{m}(T)$ (see text for details). d) The Luttinger parameter *K* calculated from $\chi^{\prime }(\mu_{0}H)$ and spinon velocity *u*, is shown as a function of applied field.

At the saturation field QCP $\mu_{0}H_{s}$, the theory given by Eq. 3 is invariant with respect to scaling transformations (see Supplementary Material, section 7 for details), resulting in magnetization and specific heat exhibiting scaling. So a plot of ($M_{s}-M$)/$\sqrt{T/J}$ as a function of $\mu_{0}(H_{s}-H)/T$ (with $\mu_{0}H_{s}$ = 1.72 T) for different values of $\mu_{0}H$ should result in a data collapse as shown in Fig. 5a. A similar plot of $C_{m}/\sqrt{T}$ as a function of $\mu_{0}(H_{s}-H)/T$ gives the data collapse for $\mu_{0}H_{s}=1.7$ T as seen in Fig. 5b, similar to the scaling of data observed in other syatems (46, 47).

The TLL universality class is characterized by collective spinon excitations that are coherent and linearly dispersing at low energies (34, 48). Consequently, the molar specific heat of a TLL at low temperatures is given by (20, 49):


(6) $$C_{TLL}=N_{A}\frac{\pi k_{B}T}{6u}$$

where $N_{A}$ is the Avogadro constant and *u* the spinon velocity. A straight line fit to the *T*-linear regime of the $C_{m}(T)$ data for $\mu_{0}H<\mu_{0}H_{s}$ (for 0 T data, the linear fit is shown as red line in the insets of Fig. 3a and b) yielded the spinon velocity in accordance with Eq. 6, as shown in Fig. 5c. It is observed that *u* decreases with increasing field, likely due to the increased presence of string-like domains of polarized spins within the system (23, 45).

Having obtained *u*, the other parameter of the field theory-Luttinger parameter *K*, was obtained from the relation between $\chi^{\prime }(\mu_{0}H)$, *u* and *K* as (20, 50):


(7) $$\chi^{\prime }(\mu_{0}H)=N_{A}\frac{(g\mu_{B}{)}^{2}}{2\pi k_{B}}\left(\frac{K}{u}\right)$$

and utilizing field-dependent $\chi^{\prime }(\mu_{0}H)$ at 0.03 K and *u* values obtained above. The calculated *K* values are plotted as a function of $\mu_{0}H$ in Fig. 5d. It can be seen that *K* is positive for all values of applied fields indicating that the interactions in TLL are repulsive in nature (20, 43, 51, 52). Furthermore, the value of *K* is 0.55 for zero field, tantalizingly close to the expected value of 0.5 (20) indicating that CuD realizes the TLL state quite well.

## Materials and methods

### Synthesis

CuD crystals were grown using liquid–liquid diffusion technique where 0.100 g (0.3 mmol) of potassium bis(oxalato)cuprate(II) dihydrate was dissolved in 12 mL of distilled water to make solution A. Similarly, 0.059 g (0.6 mmol) of 4-aminopyridine was dissolved in 12 mL of ethyl acetate to make the less dense solution B. Solution A was added to the bottom of a cylindrical glass vessel to create the bottom layer. The top layer was created using solution B that was added to the cylinder slowly creating a liquid/liquid boundary. Large sized single crystals of CuD formed at the bottom of the cylinder after 2 months.

### Measurements

Single-crystal X-ray diffraction (SCXRD) measurements were done on a Bruker APEX II CCD diffractometer using graphite-monochromatized Mo-K*α* radiation (λ=0.711Å) at a room temperature of 296(2) K. DC magnetic measurement were performed on a single-crystal of CuD having a mass of 14 mg in the temperature range of 1.8< T <150 K using a Quantum Design SQUID (Superconducting Quantum Interference Device) magnetometer (Model MPMS3). Magnetization measurements in the temperature range of 0.38< T <2 K were done using a ^3^He insert attached to the MPMS3 (Model iHelium3). Magnetic susceptibility measurements were conducted using a custom-built ac susceptometer within a temperature range of 0.03 K to 0.8 K. An ac magnetic field with a magnitude of 1.2 Oe and a frequency of 471 Hz was generated by an ac current source (Stanford Research, CS 580), while a lock-in amplifier (Stanford Research, SR 830) was utilized to record the corresponding ac signal. The sample temperature was monitored by a calibrated $RuO_{2}$ sensor positioned adjacent to the ac susceptometer. The magnetic susceptibility measured with the ac susceptometer can be considered equivalent to the under dc conditions, as the frequency of the ac magnetic field falls within the dc limits for the sample. The specific heat data were collected on a 0.85 mg single crystal in the temperature range 0.09< T <4 K using a Quantum Design PPMS equipped with a ^3^He–^4^He dilution refrigerator.

### Theoretical models

The thermodynamic properties of the integrable spin-$\frac{1}{2}$ AfHc were obtained through a combination of the quantum transfer matrix method and the Bethe Ansatz by Klumper (18) by considering a linear energy-momentum dispersion of the spinons having a velocity v=πJ. A set of nonlinear integral equations were derived, the solutions of which determine the free energy. We have computed the derivatives of the free energy equations (analytically), which have been used to obtain the equations for specific heat, magnetization and susceptibility. An efficient iterative scheme utilizing fast Fourier transform has been implemented for solving these equations numerically over a wide range of temperatures and fields (see Supplementary Material, section 6 for details of the derivations and the numerical implementation).

The effective spinless fermion field theory that describes the QCP near the saturation field starts with the Jordan–Wigner transformation for the spin chain to arrive at a spinless fermionic form. The Heisenberg model $H=J\sum_{i}S_{i}\cdot S_{i+1}+\sum_{i}g\mu_{B}H\cdot S_{i}$ with $H=H\hat{z}$ gives the polarized state |…↓↓↓↓…⟩ above the saturation field $H_{s}$. Taking it as the fermionic vacuum, the mapping is $S_{i}^{+}=\prod_{k<i}(-1{)}^{n_{k}}c_{i}^{†}$, $S_{i}^{-}=\prod_{k<i}(-1{)}^{n_{k}}c_{i}$ and $S_{i}^{z}=c_{i}^{†}c_{i}-\frac{1}{2}=n_{i}-\frac{1}{2}$ where i∈Z tracks sites on the spin chain. This leads to the fermionic Hamiltonian, $\sum_{i}(\frac{J}{2}(c_{i}^{†}c_{i+1}+c_{i+1}^{†}c_{i})+Jn_{i}n_{i+1})+\sum_{i}((J_{H}-J)n_{i}+(J-2J_{H})/4)$ where $J_{H}=g\mu_{B}H$. A continuum field ψ(x) is built out of the lattice fermion degrees of freedom by setting $\psi (x_{i})=(-1{)}^{i}c_{i}/\sqrt{a}$ where *a* is the lattice parameter between two neighboring spins. Doing a gradient expansion $\psi (x_{i+1})=\psi (x_{i}+a)∼\psi_{(}x_{i})+a\partial_{x}\psi (x)+\frac{a^{2}}{2}\partial_{x}^{2}\psi (x)\ldots$, one arrives at the continuum theory


(8) $$\int dx\left(Ja^{2}\psi^{†}(x)\partial_{x}^{2}\psi (x)-(2J-J_{H})\psi^{†}(x)\psi (x)\right)$$

for the quadratic terms, and


(9) $$\int dx\left(-Ja^{3}\psi^{†}(x)\partial_{x}\psi^{†}(x)\psi (x)\partial_{x}\psi (x)\right)$$

for the interaction term. A contact term $\propto \int dx(\psi^{†}(x)\psi (x){)}^{2}$ is absent due to the spinless nature of the fermions.

## Conclusion

To conclude, we show the first experimental phase diagram of a spin-$\frac{1}{2}$ antiferromagnetic Heisenberg chain by utilizing Wilson ratio in single crystals of a new metal-organic compound $C_{14}H_{18}CuN_{4}O_{10}$ depicting Tomonaga–Luttinger liquid, field-induced quantum critical and FP phases. The construction of the phase diagram over large range of fields and temperatures, and identification of different phases was enabled by the unique low energy scale of the exchange interactions in $C_{14}H_{18}CuN_{4}O_{10}$. Wilson ratio was calculated using magnetization, magnetic susceptibility and specific heat measurements in $C_{14}H_{18}CuN_{4}O_{10}$. Theoretical magnetization and specific heat curves generated using quantum transfer matrix method were found to match the experimentally obtained data very well. Quantum critical phase boundaries were found to affect a large portion of the phase diagram. Consequently, quantum critical scaling obtained through field theoretical methods was shown to hold true over large areas of phase diagram through data collapse in magnetization and specific heat. Finally, parameters of the Tomonaga–Luttinger liquid theory, namely, spinon velocity and Luttinger parameter were calculated was found to match with theoretical predictions very well. In order to probe the transformation of the spinon excitation spectrum (corresponding to the TLL state) to that of the magnon spectrum of the FP state, we intend to perform inelastic neutron scattering measurements on CuD and construct a magnetic phase diagram. Such measurements would be facilitated by the availability of large sized single crystals of CuD as well as the low saturation field $\mu_{0}H_{s}$ of ∼ 1.7 T easily accessible in various neutron facilities worldwide.

## Supplementary Material

pgae363_Supplementary_Data

## Data Availability

All study data are included in the article and/or Supplementary Material, Appendix.
