# Supplementary material for: Tomonaga–Luttinger liquid and quantum criticality in spin-½ antiferromagnetic Heisenberg chain C14H18CuN4O10 via Wilson ratio
Source: PNAS Nexus. 2024 Aug 23;3(9):pgae363. doi: 10.1093/pnasnexus/pgae363 (PMC11391949; doi:10.1093/pnasnexus/pgae363)
Supplement: pgae363_Supplementary_Data [file pgae363_supplementary_data.pdf]

PNAS nexus

Supporting Information for

Tomonaga-Luttinger liquid and quantum criticality in spin-$\frac{1}{2}$ antiferromagnetic Heisenberg chain $C_{14}H_{18}CuN_4O_{10}$ via Wilson ratio

Sharath Kumar Channarayappa, Sankalp Kumar, N. S. Vidhyadhiraja, Sumiran Pujari, M. P. Saravanan, Amal Sebastian, Eun Sang Choi, Shalinee Chikara, Dolly Nambi, Athira Suresh, Siddhartha Lal, D. Jaiswal-Nagar

D. Jaiswal-Nagar.
E-mail: deepshikha@iisertvm.ac.in

This PDF file includes:

Figs. S1 to S8
SI References

1. Crystal structure

In Fig. S1, the structure of $C_{14}H_{18}CuN_4O_{10}$ features corner-sharing CuO_6 octahedra that form Cu–Cu chains along the a-axis. These chains are well isolated in the b and c directions.

2. Magnetisation

The exchange coupling constant J/k_B between Cu^{2+} ions was determined by fitting the temperature dependent magnetisation data to the following equation:

$$M(T)/H = \chi_0 + \frac{C_{para}}{T} + \chi_{spin}(T) \qquad [1]$$

where χ_0 represents the diamagnetic contribution arising due to closed shell structure of the atoms; $\frac{C_{para}}{T}$ the paramagnetic contribution arising due to impurities, and $\chi_{spin}(T)$ the spin susceptibility. The high-temperature series expansion equation of magnetic susceptibility of a spin-$\frac{1}{2}$ uniform AfHc is given by (1):

$$\chi_{spin}(T) = \frac{N\mu_B^2 g^2}{k_B T} \frac{0.25 + 0.0775x + 0.0752x^2}{1 + 0.993x + 0.1721x^2 + 0.7578x^3} \qquad [2]$$

where the symbols have their usual meanings and $x = J/k_B T$. In Fig. S2, the best fit of eq. 1 to the experimental data is shown as a red solid curve. The fit parameters obtained were $\chi_0 = -4.16 \times 10^{-4}$, paramagnetic impurity concentration $C_{para} = 0.33$, intra-coupling constant $J/k_B = 1.23$ K and Landé factor $g = 2.03$.

3. Specific heat and Debye-Einstien model

In order to evaluate the magnetic component of the specific heat, $C_m(T)$, the phonon contribution to the specfic heat, C_p, needs to be subtracted from the total specific heat C_v obtained experimentally and shown in Fig. S3. This was done by fitting C_v to the Debye-Einstein model using a linear combination of one Debye and two Einstein terms as shown in the equation below (2):

$$C_p(T) = mC_D(\theta_D, T) + \sum_{i=1}^{2} t_i C_{E_i}(\theta_{E_i}, T) \qquad [3]$$

where m and t_i represent the weightage of the Debye and Einstien terms respectively. C_D and C_E are the Debye and Einstein contributions to the C_p, as given by the following equations:

$$C_D(T) = 9nR\left(\frac{T}{\theta_D}\right) \int_0^{\frac{\theta_D}{T}} \frac{X^4 e^x}{(e^x - 1)^2} dx \qquad [4]$$

$$C_E(T) = 3nR\left(\frac{\theta_E}{T}\right)^2 \frac{e^{\frac{\theta_E}{T}}}{(e^{\frac{\theta_E}{T}} - 1)^2} \qquad [5]$$

where n, R, θ_D and θ_E are the number of atoms per formula unit, universal gas constant, Debye temperature and Einstein temperature, respectively. The fitted curve is shown in Fig. S3 as a red solid curve. The obtained fitting parameters are $\theta_D = 136.96$ K, $\theta_{E_1} = 247.97$ K, $\theta_{E_2} = 4548.13$ K, $m = 0.115$, $t_1 = 0.132$, and $t_2 = 0.782$.

4. Coefficient of magnetic contribution to specific heat

In Fig. S4, $C_m(T)/T$ is plotted as a function of temperature for various magnetic field values. The peak temperature (T_p) is marked by an arrow and shown as open circles in Fig. 1 of the main article. T_p is found to decrease steadily with an increase in the applied magnetic field until 1.2 T above which it falls below the lowest measurable temperature of 0.1 K. At fields near $\mu_0 H_s$, $C_m(T)/T$ exhibits divergence as $T \to 0$ K, indicating quantum criticality (3, 4). For fields above $\mu_0 H_s$, T_p appears above 2 T and moves to higher temperatures with further increase in field.

5. Wilson ratio

The Wilson ratio, R_w, is estimated by using the formula:

$$R_w = \frac{4}{3}\left(\frac{\pi k_B}{\mu_B g}\right)^2 \frac{\chi'}{C_m/T} \qquad [6]$$

where the symbols have their usual meanings. χ represents the magnetic susceptibility while C_m/T denotes the coefficient of the magnetic contribution to specific heat. In Fig. S5, the estimated Wilson ratio is plotted as a function of field for different values of temperature. The fields corresponding to the maximum value of R_w are marked as open stars in Fig. 1 of the main article.

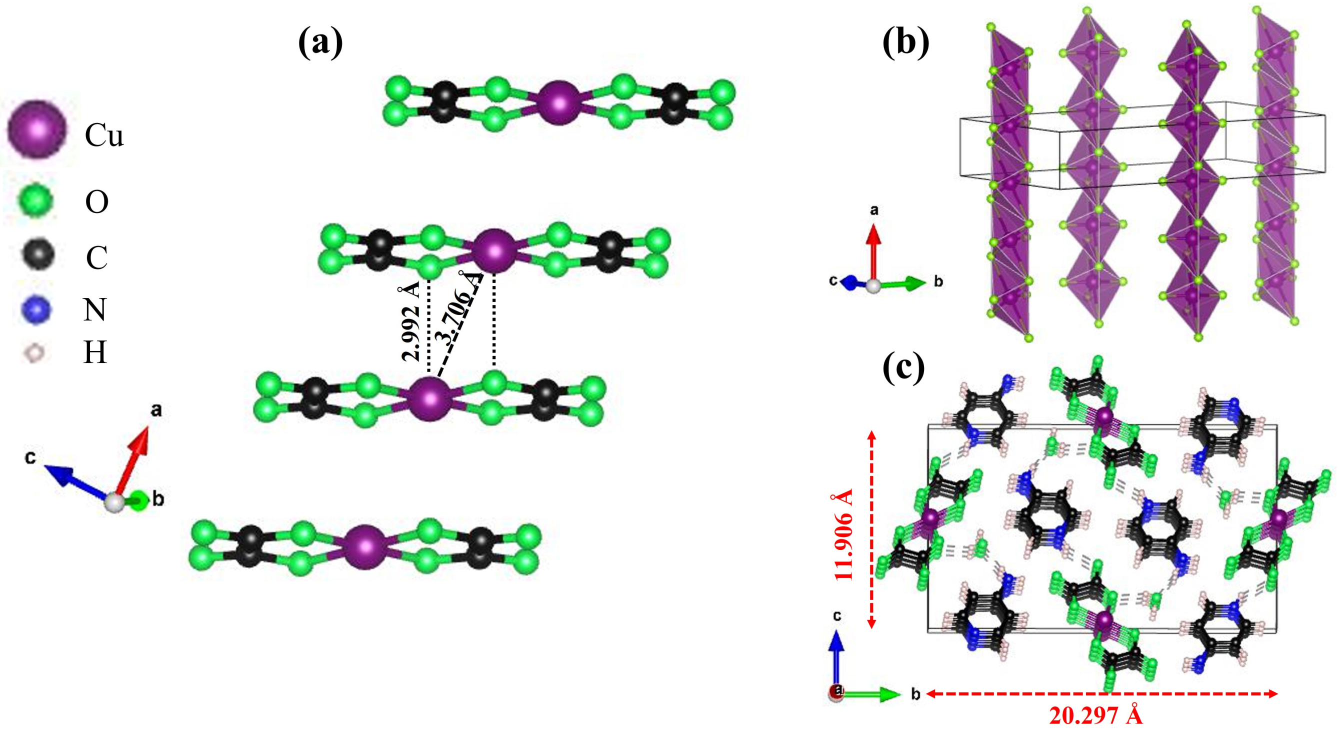

Fig. S1. (a) Chain structure formation in $C_{14}H_{18}CuN_4O_{10}$ along the a-axis through stacking of $[Cu(C_2O_4)_2]^{-2}$ units. (b) Infinite chains of $Cu^{2+}O_6$ octahedra sharing common apical oxygen atoms. (c) bc plane of $C_{14}H_{18}CuN_4O_{10}$ with Cu–Cu distances of 20.297 Å along the b-axis and 11.906 Å along the c-axis.

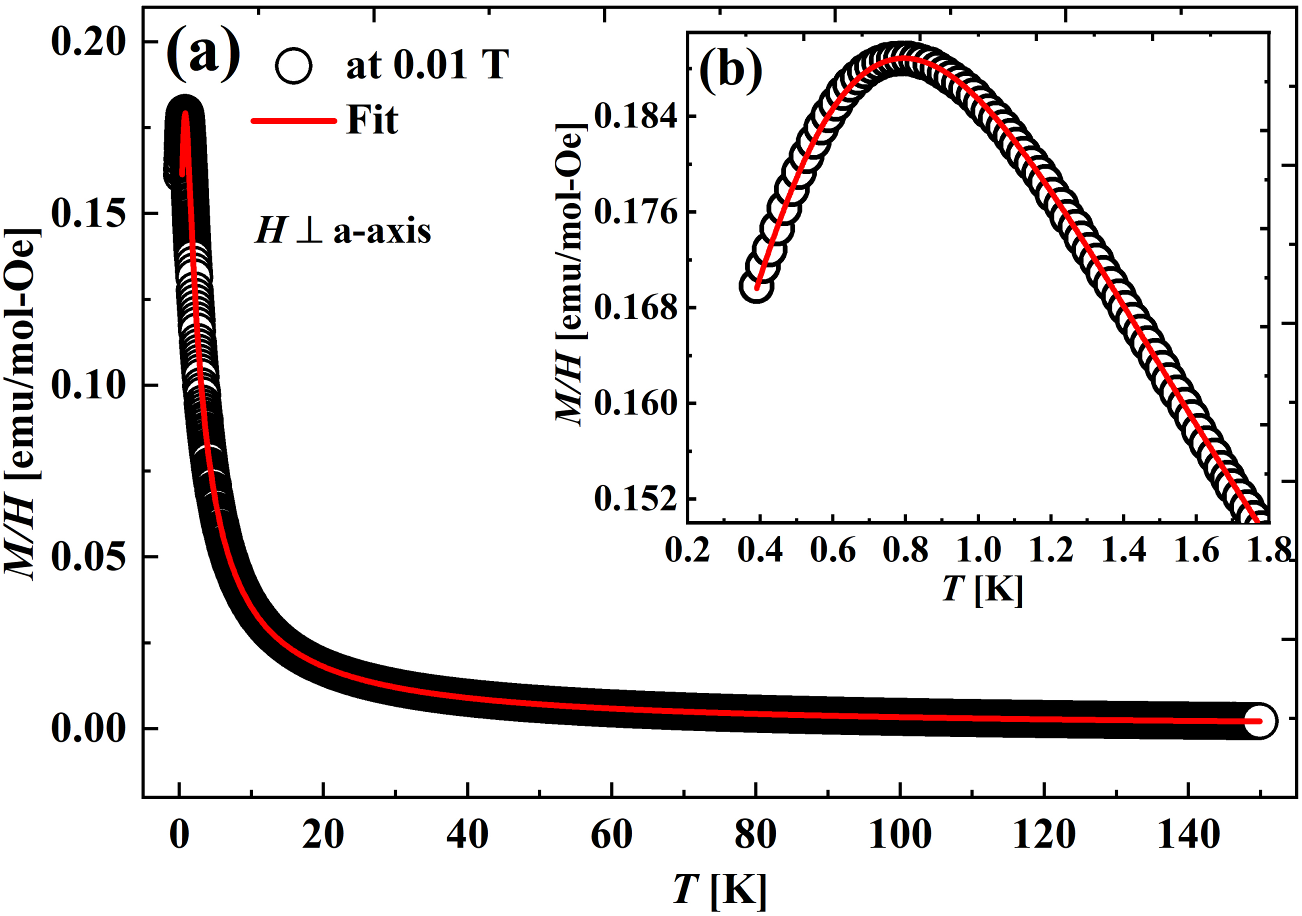

Fig. S2. (a) Temperature variation of magnetisation at an applied field of 0.01 T in $C_{14}H_{18}CuN_4O_{10}$. Open circles represent the data points, while the red solid curve is a fit to the uniform spin-$\frac{1}{2}$ AfHc model. (b) M-T data shown in the lower temperature range of 0.4 K to 1.8 K to show the peak in (a) on an expanded scale.

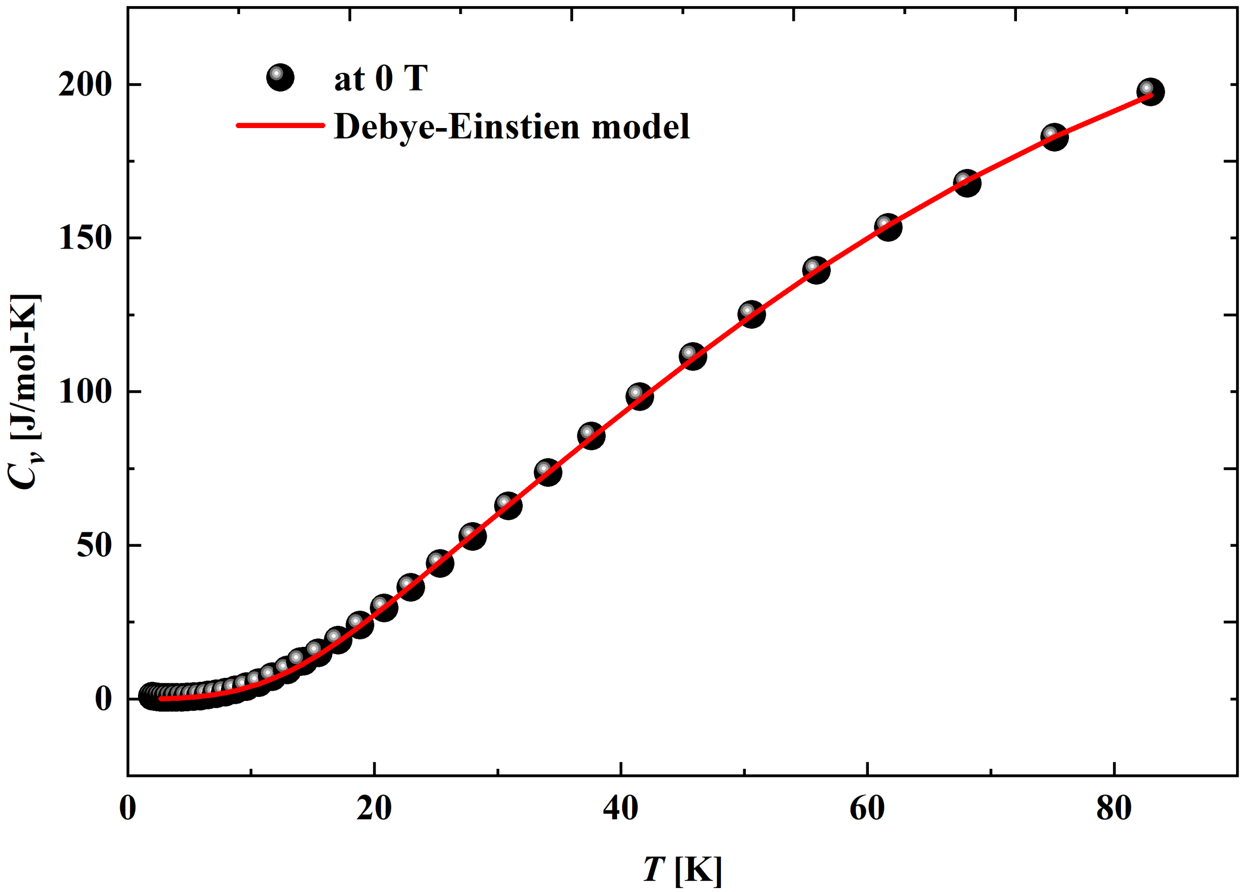

Fig. S3. Temperature variation of specific heat in $C_{14}H_{18}CuN_4O_{10}$ in the temperature range of 2 K to 80 K. Solid spheres represent the zero field data while the red solid curve is a fit to the Debye-Einstein model.

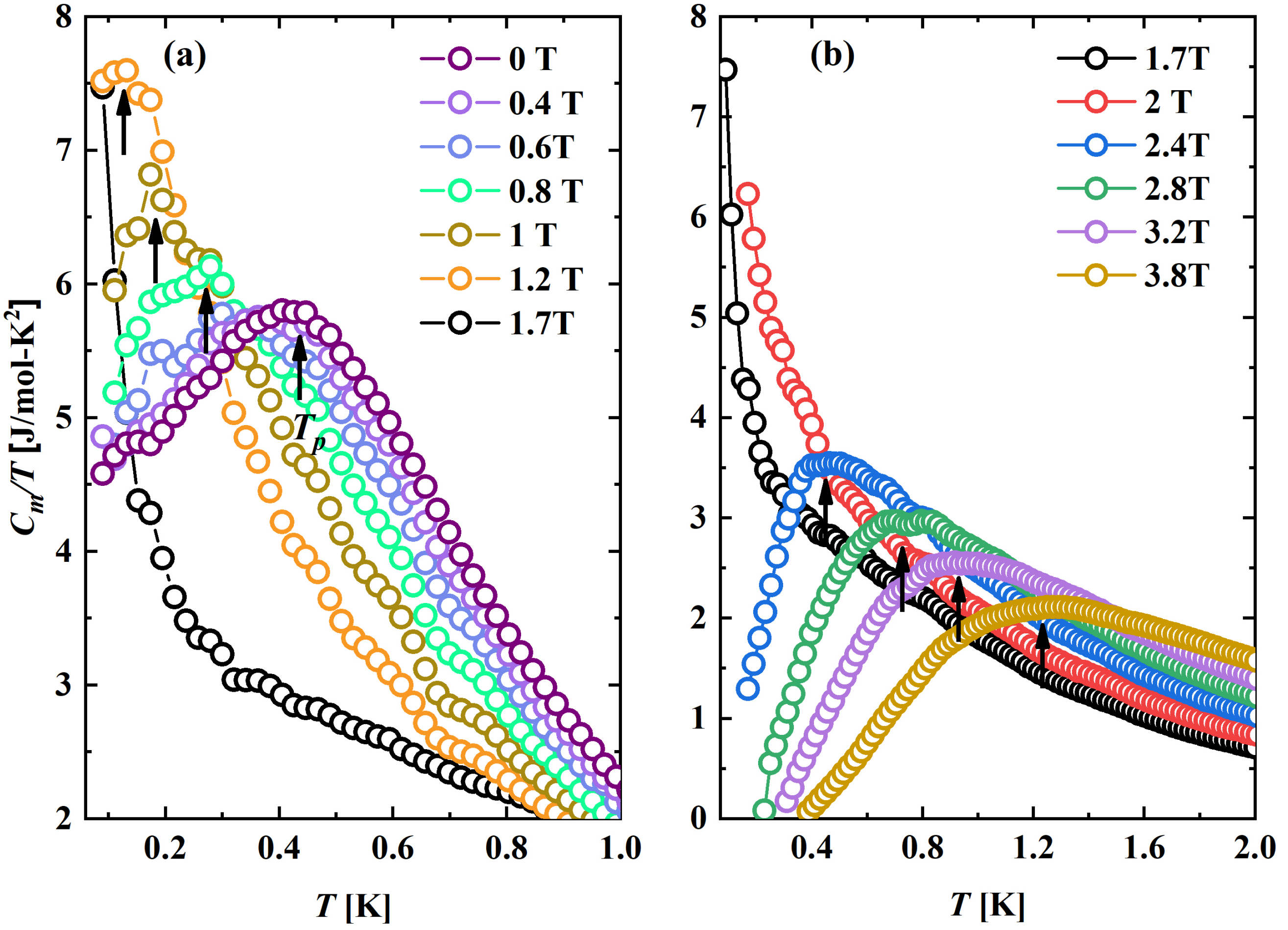

Fig. S4. The magnetic contribution to specific heat divided by temperature in $C_{14}H_{18}CuN_4O_{10}$ is plotted as a function of temperature for various field values until (a) saturation field $\mu_0 H_s$ (b) above $\mu_0 H_s$.

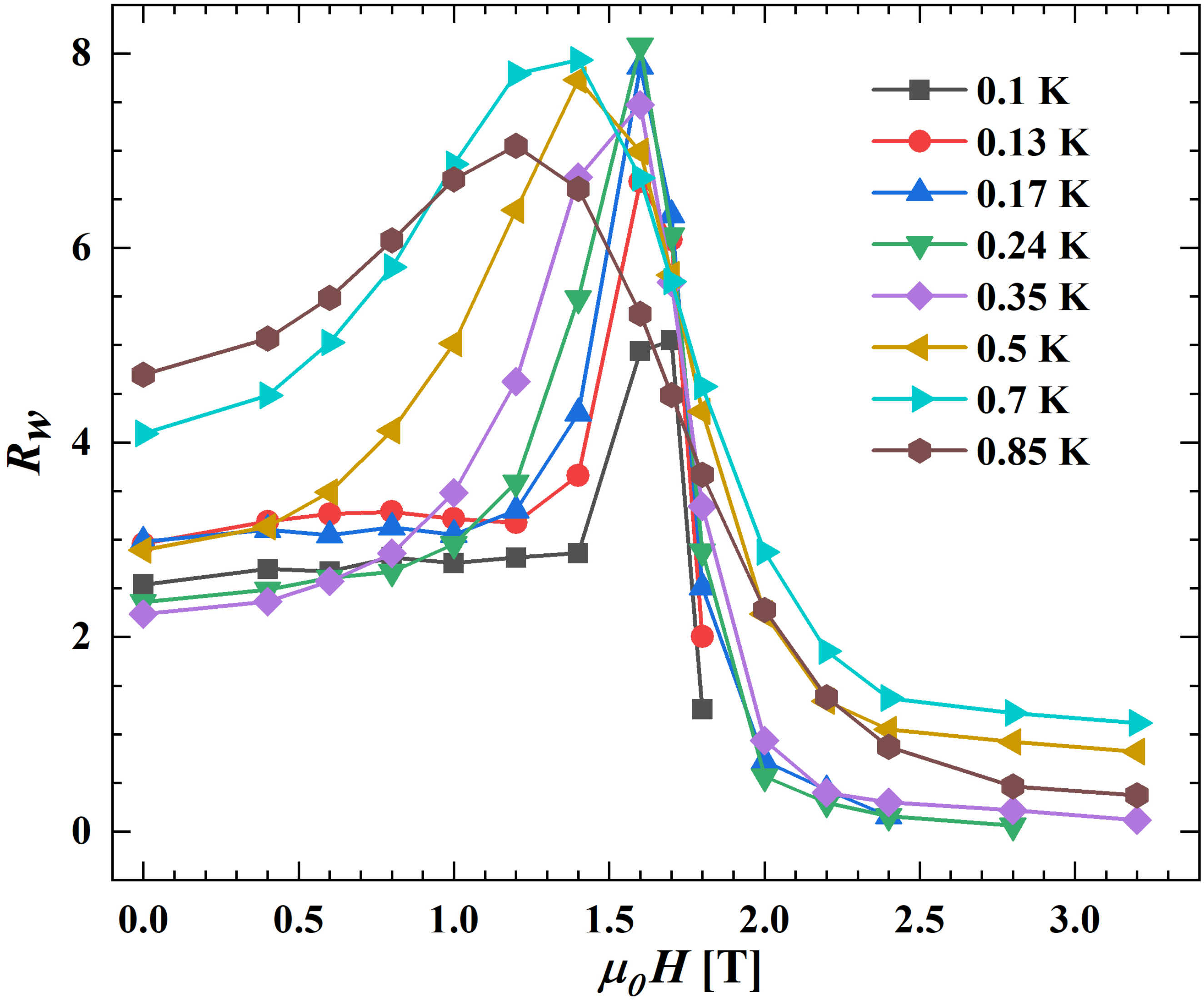

Fig. S5. Wilson ratio in $C_{14}H_{18}CuN_4O_{10}$ is plotted as a function of magnetic field for different values of temperature.

6. XXX Model

A. Introduction. Using the quantum transfer matrix approach and the Bethe ansatz, a set of equations to compute free energy for the uniform and the anisotropic one-dimensional spin-$\frac{1}{2}$ Heisenberg model were derived by Klumper(3, 5, 6). The free energy per lattice site of the spin-$\frac{1}{2}$, uniform, 1-d AfHc, with exchange coupling J, is expressed in terms of auxiliary functions, $\mathfrak{U}(x)$ and $\bar{\mathfrak{U}}(x)$ as (3):

$$\beta f = \beta e_0 - \frac{1}{2\pi}\int_{-\infty}^{+\infty} \frac{log[\mathfrak{U}\bar{\mathfrak{U}}(x)]}{cosh(x)} dx\,.$$

The auxiliary functions are, in turn, given by the following set of non-linear integral equations:

$$log\mathfrak{a}(x) = \frac{-\nu\beta}{coshx} + \phi + \int_{-\infty}^{+\infty} [K(x-y)log\mathfrak{U}(y) - K(x-y-i\pi+i\epsilon)log\bar{\mathfrak{U}}(y)]dy$$

$$log\bar{\mathfrak{a}}(x) = \frac{-\nu\beta}{coshx} - \phi + \int_{-\infty}^{+\infty} [K(x-y)log\bar{\mathfrak{U}}(y) - K(x-y+i\pi-i\epsilon)log\mathfrak{U}(y)]dy$$

where $\mathfrak{U}(x) = 1+\mathfrak{a}$, and $\bar{\mathfrak{U}}(x) = 1+\bar{\mathfrak{a}}$; $v = \pi J$; $\phi = \frac{\beta}{2}h$, with $h = g\mu_B H$ and H is the applied magnetic field. $K(x)$ is the integration kernel given by

$$K_1(x) = \frac{1}{\pi}\int_0^{\infty} \frac{e^{-\pi k}}{\cosh(\pi k)} \cos(2kx)dk$$

The Integral is computed and the plot of the function is shown below

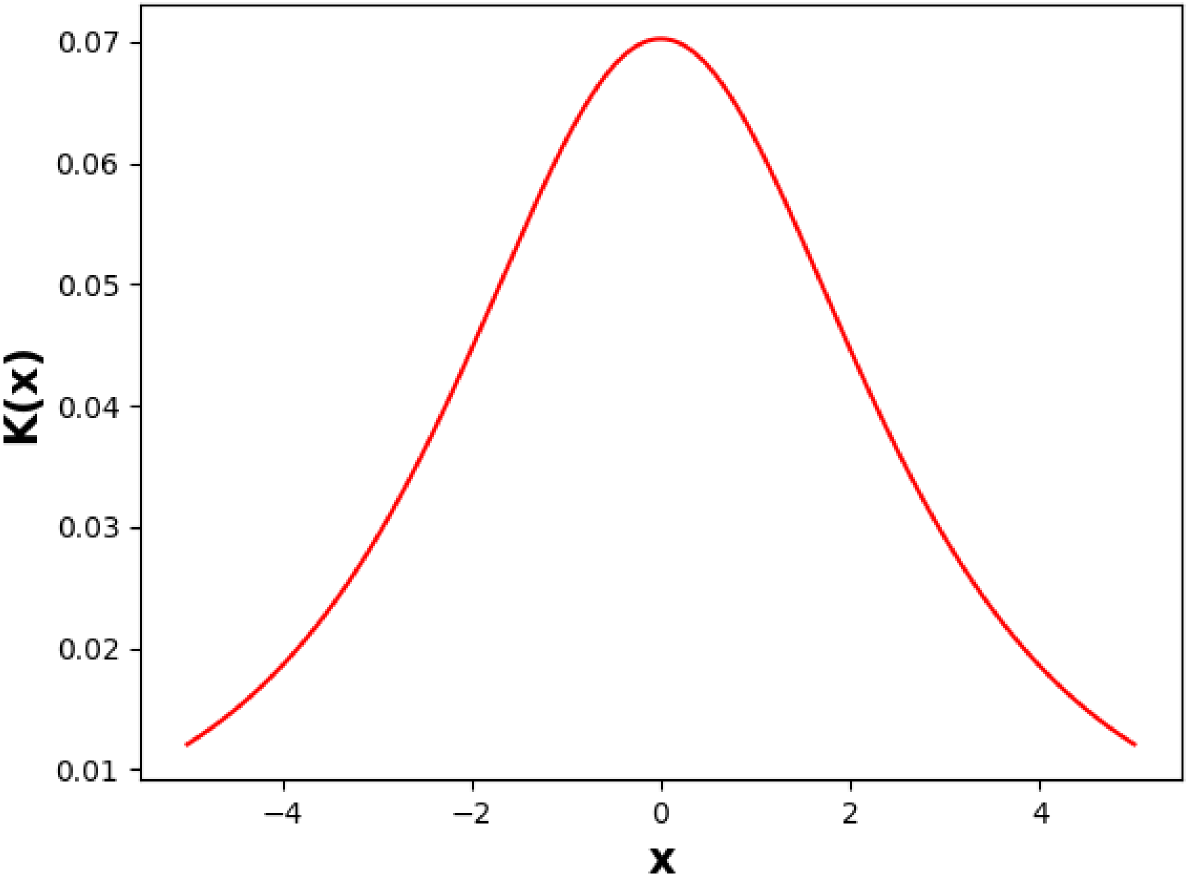

Fig. S6. Plot of K(x)

The equation for the Integration Kernel, K(x-iπ+iϵ) is given below,

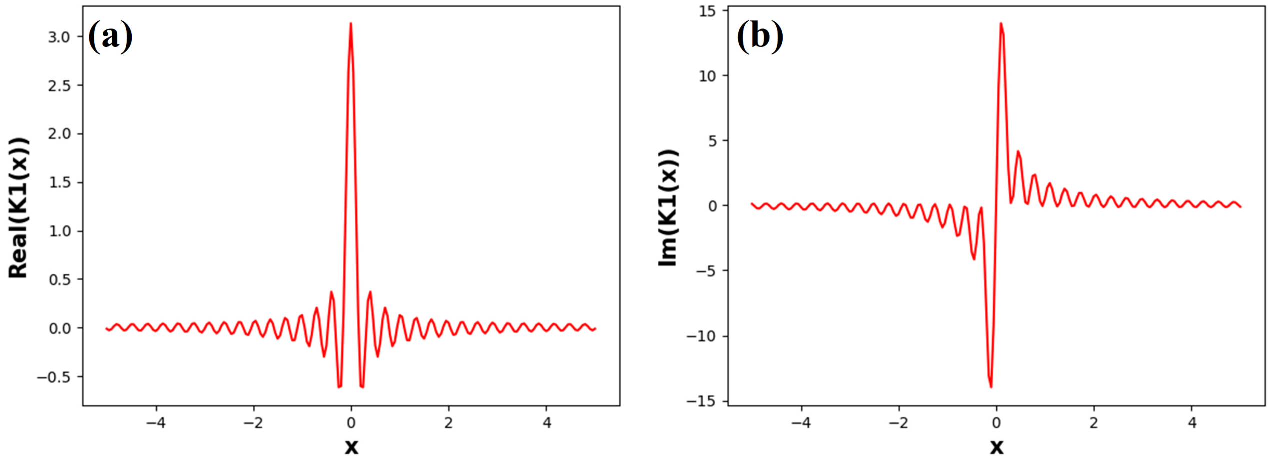

Fig. S7. (a) Re(K_1(x)). (b) Im(K_1(x))

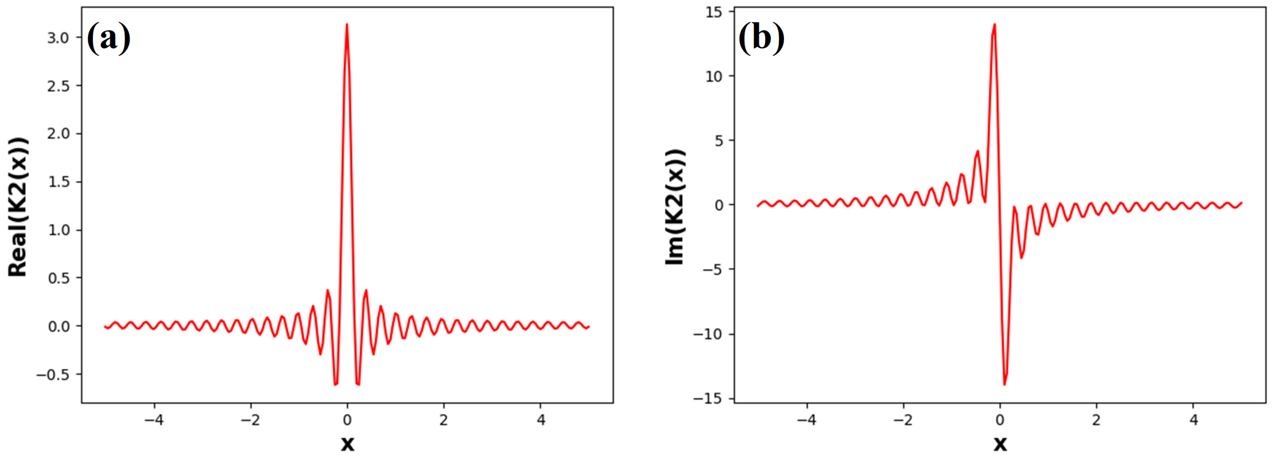

Fig. S8. (a) Re(K_2(x)). (b) Im(K_2(x))

$$K_2(x) = K_1(x - i\pi + i\epsilon) = \frac{1}{2\pi}\int_0^{\infty} \frac{e^{\frac{-\pi k}{2}}}{\cosh(\frac{\pi k}{2})} \cos(kx - i\pi + i\epsilon)dk$$

The Integral is computed and the plot of the function is shown below
The equation for the Integration Kernel, K(x+$i\pi$-$i\epsilon$) is given below,

$$K_3(x) = K_1(x + i\pi - i\epsilon) = \frac{1}{2\pi}\int_0^{\infty} \frac{e^{\frac{-\pi k}{2}}}{\cosh(\frac{\pi k}{2})} \cos(kx + i\pi - i\epsilon)dk$$

For $\epsilon \to 0$

B. Numerical implementation of XXX Model.

B.1. Integration Kernels.

$$K_1(x) = \frac{1}{2\pi}\int_0^{\infty} \frac{e^{\frac{-\pi k}{2}}}{\cosh(\frac{\pi k}{2})} \cos(kx)dk$$

$$K_2(x) = K_1(x - i\pi + i\epsilon) = \frac{1}{2\pi}\int_0^\infty \frac{e^{\frac{-\pi k}{2}}}{\cosh(\frac{\pi k}{2})}\cos(kx - i\pi + i\epsilon)dk$$

$$K_2(x) = \frac{1}{2\pi}\int_0^\infty \frac{e^{\frac{-\pi k}{2}}}{\cosh(\frac{\pi k}{2})}[coskxcosh(k(\pi - \epsilon)) + isinkxsinh(k(\pi - \epsilon))]dk$$

$$K_3(x) = [K_2(x)]^*$$

To avoid numerical overflow we do the following,

$$\frac{e^{\frac{-\pi k}{2}}}{\cosh(\frac{\pi k}{2})} = \frac{2e^{\frac{-\pi k}{2}}}{e^{\frac{\pi k}{2}} + e^{\frac{-\pi k}{2}}} = \frac{2e^{-\pi k}}{1 + e^{-\pi k}}$$

$$\frac{e^{\frac{-\pi k}{2}}}{\cosh(\frac{\pi k}{2})}cosh(k(\pi - \epsilon)) = \frac{e^{-\pi k}}{1 + e^{-\pi k}}\left(e^{k(\pi-\epsilon)} + e^{-k(\pi-\epsilon)}\right) = \frac{e^{-k\epsilon} + e^{-k(2\pi-\epsilon)}}{1 + e^{-\pi k}}$$

$$\frac{e^{\frac{-\pi k}{2}}}{\cosh(\frac{\pi k}{2})}sinh(k(\pi - \epsilon)) = \frac{e^{-\pi k}}{1 + e^{-\pi k}}\left(e^{k(\pi-\epsilon)} - e^{-k(\pi-\epsilon)}\right) = \frac{e^{-k\epsilon} - e^{-k(2\pi-\epsilon)}}{1 + e^{-\pi k}}$$

At $\epsilon \to 0$,

$$\frac{e^{\frac{-\pi k}{2}}}{\cosh(\frac{\pi k}{2})}cosh(k(\pi - \epsilon)) = \frac{1 + e^{-2\pi k}}{1 + e^{-\pi k}}$$

$$\frac{e^{\frac{-\pi k}{2}}}{\cosh(\frac{\pi k}{2})}sinh(k(\pi - \epsilon)) = \frac{1 - e^{-2\pi k}}{1 + e^{-\pi k}}$$

B.2. Derivatives Implementation for specific heat computation. Let us define the convolution function,$\mathfrak{C}(g(x), h(x))$ as shown below

$$\mathfrak{C}(g(x), h(x)) = \int_{-\infty}^{\infty} g(\tau)h(x - \tau)d\tau$$

Using the above definition the equations are

$$log\mathfrak{a}(x) = \frac{-\nu\beta}{coshx} + \phi + \mathfrak{C}(K(x), log\mathfrak{U}(x)) - \mathfrak{C}(K(x - i\pi + i\epsilon), log\bar{\mathfrak{U}}(x))$$

$$log\mathfrak{a}(x) = \frac{-\nu\beta}{coshx} + \phi + \mathfrak{C}(K(x), log\bar{\mathfrak{U}}(x)) - \mathfrak{C}(K(x + i\pi - i\epsilon), log\mathfrak{U}(x))$$

Multiplying by $k_b T/J = \bar{T}$

$$\bar{T}log\mathfrak{a}(x) = \frac{-\pi}{coshx} + \frac{h}{2} + \mathfrak{C}(\bar{T}log\mathfrak{U}(x), K_1(x)) - \mathfrak{C}(\bar{T}log\bar{\mathfrak{U}}(x), K_2(x))$$

$$\bar{T}log\mathfrak{a}(x) = \frac{-\pi}{coshx} - \frac{h}{2} + \mathfrak{C}(\bar{T}log\bar{\mathfrak{U}}(x), K_1(x)) - \mathfrak{C}(\bar{T}log\mathfrak{U}(x), K_3(x))$$

Let us define $\bar{T}log\mathfrak{U}(x) = L_u$ $\quad \bar{T}log\bar{\mathfrak{U}}(x) = L_{\bar{u}}$ $\quad \bar{T}log\mathfrak{a}(x) = L_a$ $\quad \bar{T}log\bar{\mathfrak{a}}(x) = L_{\bar{a}}$
Then the equations become,

$$\boxed{L_a = \frac{-\pi}{coshx} + \frac{h}{2} + \mathfrak{C}(L_u, K_1) - \mathfrak{C}(L_{\bar{u}}, K_2)}$$

$$\boxed{L_{\bar{a}} = \frac{-\pi}{coshx} - \frac{h}{2} + \mathfrak{C}(L_{\bar{u}}, K_1) - \mathfrak{C}(L_u, K_3)}$$

We know that,

$$\mathfrak{U} = 1 + \mathfrak{a}$$

$$\bar{T}log\mathfrak{U} = \bar{T}log(1 + \mathfrak{a})$$

$$\boxed{L_u = \bar{T}log\left(1 + exp\left(\frac{L_a}{T}\right)\right)}$$

Similarly,

$$\boxed{L_{\bar{u}} = \bar{T}log\left(1 + exp\left(\frac{L_{\bar{a}}}{T}\right)\right)}$$

B.3. First Derivatives. Taking the first derivatives of the boxed equations we get,

$$\frac{dL_a}{d\bar{T}} = \mathfrak{C}(\frac{dL_u}{d\bar{T}}, K_1) - \mathfrak{C}(\frac{dL_{\bar{u}}}{d\bar{T}}, K_2)$$

$$\frac{dL_{\bar{a}}}{d\bar{T}} = \mathfrak{C}(\frac{dL_{\bar{u}}}{d\bar{T}}, K_1) - \mathfrak{C}(\frac{dL_u}{d\bar{T}}, K_3)$$

$$T\frac{dL_a}{d\bar{T}} = L_u + exp\left(\frac{L_a - L_u}{\bar{T}}\right)\left(\bar{T}\frac{dL_a}{dT} - L_a\right)$$

$$T\frac{dL_{\bar{a}}}{d\bar{T}} = L_{\bar{u}} + exp\left(\frac{L_{\bar{a}} - L_{\bar{u}}}{\bar{T}}\right)\left(\bar{T}\frac{dL_{\bar{a}}}{dT} - L_{\bar{a}}\right)$$

B.4. Second Derivatives. Taking the second derivatives of the above equations,

$$\frac{d^2L_a}{d\bar{T}^2} = \mathfrak{C}(\frac{d^2L_u}{d\bar{T}^2}, K_1) - \mathfrak{C}(\frac{dL_{\bar{u}}^2}{d\bar{T}^2}, K_2)$$

$$\frac{d^2L_{\bar{a}}}{d\bar{T}^2} = \mathfrak{C}(\frac{dL_{\bar{u}}^2}{d\bar{T}^2}, K_1) - \mathfrak{C}(\frac{dL_u^2}{d\bar{T}^2}, K_3)$$

$$\bar{T}^3\frac{d^2L_a}{d\bar{T}^2} = \left(\bar{T}\frac{dL_u}{d\bar{T}} - L_u\right)\left[\frac{\bar{T}^3\frac{d^2L_a}{dT^2}}{T\frac{dL_a}{dT} - L_a} + T\frac{dL_a}{dT} - L_a - T\frac{dL_u}{dT} - L_u\right]$$

$$\bar{T}^3\frac{d^2L_{\bar{a}}}{d\bar{T}^2} = \left(\bar{T}\frac{dL_{\bar{u}}}{d\bar{T}} - L_{\bar{u}}\right)\left[\frac{\bar{T}^3\frac{d^2L_{\bar{a}}}{dT^2}}{\left(T\frac{dL_{\bar{a}}}{dT} - L_{\bar{a}}\right)} + \left(T\frac{dL_{\bar{a}}}{dT} - L_{\bar{a}}\right) - \left(T\frac{dL_{\bar{u}}}{dT} - L_{\bar{u}}\right)\right]$$

These equations are iteratively implemented to find L_a, $\frac{dL_a}{dT}$, $\frac{d^2L_a}{dT^2}$ and $L_{\bar{a}}$, $\frac{dL_{\bar{a}}}{dT}$, $\frac{d^2L_{\bar{a}}}{dT^2}$ and L_a, $\frac{dL_u}{dT}$, $\frac{d^2L_u}{dT^2}$ and $L_{\bar{u}}$, $\frac{dL_{\bar{u}}}{dT}$, $\frac{d^2L_{\bar{u}}}{dT^2}$.

B.5. Specific heat. We know that the equation for free energy is given by the equation,

$$\beta f = \beta e_0 - \frac{1}{2\pi}\int_{-\infty}^{+\infty}\frac{log[\mathfrak{U}\bar{\mathfrak{U}}(x)]}{cosh(x)}dx$$

$$f/J = e_0 - \frac{T'}{2\pi}\int_{-\infty}^{+\infty}\frac{log[\mathfrak{U}\bar{\mathfrak{U}}(x)]}{cosh(x)}dx$$

$$f/J = e_0 - \frac{1}{2\pi}\int_{-\infty}^{+\infty}\frac{dx}{coshx}\left[L_u + L_{\bar{u}}\right]$$

$$\boxed{C_v = \frac{1}{2\pi}\int_{-\infty}^{+\infty}\frac{dx}{coshx}\left[\bar{T}\frac{d^2L_u}{dT^2} + \bar{T}\frac{d^2L_{\bar{u}}}{dT^2}\right]}$$

B.6. Derivatives implementation for susceptibility. The first set of equations determining C_v are the ones for free energy for a given (T,h). These equations remain the same. Only the derivatives change. So we will focus only on the derivatives.

B.7. First derivatives.

$$\frac{dL_a}{dh} = \frac{1}{2} + \mathfrak{C}(\frac{dL_u}{dh}, K_1) - \mathfrak{C}(\frac{dL_{\bar{u}}}{dh}, K_2)$$

$$\frac{dL_{\bar{a}}}{dh} = -\frac{1}{2} + \mathfrak{C}(\frac{dL_{\bar{u}}}{d\bar{T}}, K_1) - \mathfrak{C}(\frac{dL_u}{dh}, K_3)$$

$$T\frac{dL_u}{dh} = exp\left(\frac{L_a - L_u}{\bar{T}}\right)\frac{dL_a}{dT}$$

$$T\frac{dL_{\bar{u}}}{d\bar{T}} = exp\left(\frac{(L_{\bar{a}} - L_{\bar{u}}}{\bar{T}}\right)\frac{dL_{\bar{a}}}{dT}$$

B.8. Second derivatives.

$$\frac{d^2L_a}{dh^2} = \mathfrak{C}(\frac{d^2L_u}{dh^2}, K_1) - \mathfrak{C}(\frac{dL^2_{\bar{u}}}{dh^2}, K_2)$$

$$\frac{d^2L_{\bar{a}}}{dh^2} = \mathfrak{C}(\frac{dL^2_{\bar{u}}}{dh^2}, K_1) - \mathfrak{C}(\frac{dL^2_u}{dh^2}, K_3)$$

$$\frac{d^2L_u}{dh^2} = exp\left(\frac{L_a - L_u}{\bar{T}}\right)\left[\frac{d^2L_a}{dh^2} + \frac{1}{T}\frac{d^2L_a}{dh^2}\left(\frac{d^2L_a}{dh^2} - \frac{d^2L_u}{dh^2}\right)\right]$$

$$\frac{d^2L_{\bar{u}}}{dh^2} = exp\left(\frac{L_{\bar{a}} - L_{\bar{u}}}{\bar{T}}\right)\left[\frac{d^2L_{\bar{a}}}{dh^2} + \frac{1}{T}\frac{d^2L_{\bar{a}}}{dh^2}\left(\frac{d^2L_{\bar{a}}}{dh^2} - \frac{d^2L_{\bar{u}}}{dh^2}\right)\right]$$

B.9. Susceptibility. Similar to the specific heat expression we can derive the susceptibility expression as the following,

$$\boxed{\chi = \frac{1}{2\pi}\int_{-\infty}^{+\infty}\frac{dx}{coshx}\left[\bar{T}\frac{d^2L_u}{dh^2} + \bar{T}\frac{d^2L_{\bar{u}}}{dh^2}\right]}$$

7. Field theory calculations

Here we give some details on the scaling forms for various thermodynamic quantities arrived from the spinless fermionic field theory written in the theoretical models section in the main text. They can be found by known methods, e.g. see Chapter 16 of Ref. (7). One can do a scaling or power counting analysis to show that the interaction term is RG-irrelevant based on dimensional grounds. This scaling argument is also described in Sec. 16.2 of Ref. (7). Thus, we can arrive at the dominant scaling behavior for the thermodynamic quantities by using the free field theory and ignoring the interaction term. The first result is for the overall free energy which takes the following scaling form:

$$\mathcal{F}(T,\mu) = (k_BT)\left(\frac{k_BT}{J}\right)^{\frac{1}{2}}\Phi\left(\frac{\mu}{k_BT}\right) = J^{-1/2}\beta^{-3/2}\Phi(\beta\mu) \qquad [7]$$

where $\Phi(x)$ is an universal scaling function, and $\mu \equiv 2J - g\mu_BH$. $\beta = (k_BT)^{-1}$. Using $C(\mu, T) = -\frac{1}{k_BT^2}\frac{\partial^2(\beta F)}{\partial\beta^2}$, we get the following scaling form for the specific heat:

$$\frac{C}{k_B} = -\frac{3}{4}\left(\frac{k_BT}{J}\right)^{1/2}\Phi\left(\frac{\mu}{k_BT}\right) + \left(\frac{\mu^2}{J(k_BT)}\right)^{1/2}\Phi'\left(\frac{\mu}{k_BT}\right) - \left(\frac{\mu^4}{J(k_BT)^3}\right)^{1/2}\Phi''\left(\frac{\mu}{k_BT}\right) \qquad [8]$$

which can be re-expressed simply as

$$\frac{C}{k_B} = \sqrt{\frac{k_BT}{J}}\Theta\left(\frac{\mu}{k_BT}\right) \qquad [9]$$

where $\Theta(x)$ is an universal scaling function. For the magnetization, one can calculate deviation from the saturation magnetization corresponding to the fully polarized state $|\ldots\downarrow\downarrow\downarrow\ldots\rangle$. This deviation becomes the spinless fermionic occupation given by $\langle N\rangle = \langle\sum_i n_i\rangle = -\frac{\partial\mathcal{F}}{\partial\mu}$, we get the following scaling form for the magnetization

$$M(H) - M_{\text{sat}} = g\mu_B\left(\frac{k_BT}{J}\right)^{\frac{1}{2}}\Phi'\left(\frac{\mu}{k_BT}\right) \qquad [10]$$

This overall scaling form can be further specified in various regimes of the phase diagram. In the fully polarized regime ($H > H_s$, $\mu < 0$, $k_BT \ll |\mu| \ll J$), we get

$$M(H) - M_{\text{sat}} = g\mu_B\left(\frac{k_BT}{4\pi J}\right)^{\frac{1}{2}}e^{\frac{\mu}{k_BT}} \qquad [11]$$

In the TLL regime ($H < H_s$, $\mu > 0$, $k_BT \ll |\mu| \ll J$), which at quadratic level is a liquid of spinons at finite density, we get

$$M(H) - M_{\text{sat}} = g\mu_B\frac{1}{4\pi(0.5)!}\left[\left(\frac{\mu}{J}\right)^{\frac{1}{2}}\left(1 - \frac{1}{24}\left(\frac{k_BT}{\mu}\right)^2 + \ldots\right)\right] \qquad [12]$$

In the QC regime ($H \sim H_s$, $|\mu| \ll k_B \ll J$) inside the "cone",

$$M(H) - M_{\text{sat}} = g\mu_B\zeta(0.5)(1 - \sqrt{2})\left(\frac{k_BT}{4\pi J}\right)^{\frac{1}{2}} \qquad [13]$$

which is now independent of μ as far as the dominant behaviour goes when contrasted with the previous limits.

References

1. JC Bonner, ME Fisher, Linear magnetic chains with anisotropic coupling. *Phys. Rev.* **135**, A640 (1964).
2. C Kittel, P McEuen, *Introduction to solid state physics.* (John Wiley & Sons), (2018).
3. A Klümper, The spin-1/2 heisenberg chain: thermodynamics, quantum criticality and spin-peierls exponents. *The Eur. Phys. J. B-Condensed Matter Complex Syst.* **5**, 677–685 (1998).
4. T Xiang, Thermodynamics of quantum heisenberg spin chains. *Phys. Rev. B* **58**, 9142 (1998).
5. A Klümper, Integrability of quantum chains: theory and applications to the spin-1/2 xxz chain. *Quantum magnetism* pp. 349–379 (2008).
6. A Klümper, Thermodynamics of the anisotropic spin-1/2 heisenberg chain and related quantum chains. *Zeitschrift für Physik B Condens. Matter* **91**, 507–519 (1993).
7. S Sachdev, *Quantum Phase Transitions.* (Cambridge University Press), 2 edition, (2011).
